# Supplementary material for: Comparison of the Fatty Acid and Triglyceride Profiles of Big Eye Tuna (Thunnus obesus), Atlantic salmon (Salmo salar) and Bighead Carp (Aristichthysnobilis) Heads
Source: Molecules. 2019 Nov 4;24(21):3983. doi: 10.3390/molecules24213983 (PMC6864674; doi:10.3390/molecules24213983)
Supplement: Supplementary file 1 [file molecules-24-03983-s001.zip › Supplementary materials/Suplementary materirals.pdf]

Table S1. Fatty acids profiles of total lipids in three fish heads (g/100g, mean ± S.D., n=3)

Fatty acid	Big eye tuna	Atlantic salmon	Big head carp
C4:0	$0.92±0.61^{a}$	$0.16±0.16^{c}$	$0.27±0.36^{b}$
C14:0	$0.94±0.85^{a}$	$0.32±0.12^{c}$	$0.59±0.30^{b}$
C15:0	$0.50±0.44^{b}$	$0.48±0.42^{b}$	$0.74±0.76^{a}$
C16:0	$3.49±0.34^{a}$	$3.82±0.21^{a}$	$2.99±1.60^{b}$
C17:0	$1.56±0.29^{a}$	$0.06±0.06^{c}$	$0.40±0.45^{b}$
C18:0	$1.80±0.27^{a}$	$0.66±0.66^{b}$	$0.34±0.49^{c}$
C20:0	$0.11±0.12^{b}$	$0.21±0.03^{a}$	$0.03±0.05^{c}$
C21:0	$0.06±0.10^{c}$	$0.79±0.79^{b}$	$1.58±2.30^{a}$
C22:0	$0.20±0.18^{b}$	$0.10±0.06^{b}$	$1.25±1.94^{a}$
C23:0	$1.06±0.40^{a}$	$0.03±0.03^{c}$	$0.68±0.41^{b}$
C24:0	$0.09±0.05^{b}$	$0.01±0.01^{b}$	$0.52±0.25^{a}$
**∑SFA**	**$10.73±3.65^{a}$**	**$6.64±2.55^{b}$**	**$9.39±8.91^{a}$**
C14:1	$0.39±0.10^{c}$	$1.28±1.25^{a}$	$0.89±0.94^{b}$
C15:1	$1.34±0.23^{a}$	$0.19±0.07^{c}$	$0.70±0.49^{b}$
C16:1	$3.03±1.14^{c}$	$17.49±8.97^{b}$	$27.42±4.36^{a}$
C17:1	$0.76±0.33^{c}$	$2.01±2.00^{a}$	$1.09±0.28^{b}$
C18:1N9	$2.78±1.79^{b}$	$5.66±1.77^{a}$	$6.68±0.72^{a}$
C20:1	$0.66±1.14^{c}$	$6.33±1.94^{a}$	$2.74±0.80^{b}$
C22:1N9	$0.45±0.62^{a}$	$0.12±0.01^{c}$	$0.58±0.69^{a}$
C24:1	$0.74±0.37^{a}$	$0.43±0.18^{b}$	$0.30±0.27^{b}$
**∑MUFA**	**$10.15±5.72^{c}$**	**$33.51±16.19^{b}$**	**$40.4±8.55^{a}$**
C18:2N6	$0.95±0.43^{c}$	$5.84±1.44^{a}$	$1.22±1.01^{b}$
C18:3N6	$0.91±0.51^{b}$	$5.42±2.01^{a}$	$0.16±0.03^{c}$
C18:3N3	$0.21±0.36^{b}$	$0.07±0.07^{c}$	$1.55±0.58^{a}$
C20:2	$0.30±0.33^{b}$	$1.78±0.65^{a}$	$0.48±0.28^{b}$
C20:3N6	$0.06±0.03^{c}$	$5.79±2.82^{a}$	$0.43±0.47^{b}$
C20:3N3	$0.61±0.68^{a}$	$0.09±0.09^{b}$	$0.59±0.23^{a}$
C20:4N6	$1.06±0.40^{a}$	$0.03±0.03^{c}$	$0.62±0.51^{b}$
C22:2	$0.31±0.11^{c}$	$1.12±0.61^{b}$	$2.24±0.42^{a}$
C20:5N3	$8.83±0.82^{a}$	$4.18±1.03^{c}$	$5.15±1.37^{b}$
C22:6N3	$36.67±1.21^{a}$	$6.39±1.3^{b}$	$3.13±1.14^{c}$
**EPA+DHA**	**$45.51±2.03^{a}$**	**$10.57±2.33^{b}$**	**$8.28±2.51^{c}$**
**∑PUFA**	**$49.91±4.88^{a}$**	**$30.71±10.05^{b}$**	**$15.57±6.04^{c}$**
**TFA**	**$70.79±14.25^{a}$**	**$70.86±28.79^{a}$**	**$65.36±23.5^{a}$**

[a-d] values in the same column with different lower-case letters are significantly different at P＜0.05.

Table S2. Fatty acids profiles of TG fraction in three fish heads. (g/100g, mean ± S.D., n=3)

Fatty Acid	Big eye tuna	Atlantic salmon	Big head Carp
C4:0	2.07±1.01[b]	20.89±7.32[a]	1.75 ±1.08[c]
C14:0	1.72±0.47[a]	0.65±0.38[b]	5.18±0.92[a]
C15:0	N. D.	0.96±1.53[b]	N. D.
C16:0	0.1±0.03[c]	4.47±0.77[a]	0.38±0.15[b]
C17:0	1.02±0.04[a]	0.03±0.00[d]	N. D.
C18:0	N. D.	2.31±0.27[b]	N. D.
C20:0	0.09±0.02[b]	0.03±0.03[b]	0.07±0.05[b]
C22:0	0.08±0.02[b]	0.03±0.00[b]	0.23±0.10[a]
C23:0	N. D.	0.01±0.01[c]	N. D.
C24:0	2.78±0.65[b]	N. D.	5.34±1.75[a]
**SFA**	**7.86±1.88[d]**	**27.04±8.57[a]**	**12.95±4.05[b]**
C14:1	0.83±0.23[a]	0.02±0.01[c]	1.68±0.48[a]
C15:1	2.69±1.96[a]	N. D.	3.62±3.86[a]
C16:1	18.21±4.62[a]	1.15±0.16[a]	16.05±11.11[a]
C17:1	2.2±0.52[b]	N. D.	2.24±1.40[a]
C18:1N9	14.85±3.62[a]	24.47±9.34[a]	16.6±4.79[a]
C20:1	0.18±0.24[b]	3.04±0.91[c]	9.26±2.30[a]
C22:1N9	0.15±0.04[a]	N. D.	0.24±0.10[a]
C24:1	N. D	0.13±0.05[b]	N. D.
**MUFA**	**38.97±10.8[a]**	**28.81±4.57[a]**	**49.69±24.04[a]**
C18:2N6	0.54±0.15[c]	11.10±1.45[a]	4.00±3.05[b]
C18:3N3	1.64±0.38[a]	1.34±0.56[a]	0.34±0.08[b]
C18:3N6	0.08±0.05[b]	N. D.	1.24±0.96[a]
C20:2	0.25±0.07[c]	0.89±0.22[a]	1.07±0.54[a]
C20:3N6	0.08±0.05[b]	0.43±0.13[b]	0.23±0.14[a]
C20:3N3	1.94±0.46[a]	0.93±0.01[a]	3.96±1.58[a]
C20:4N6	N. D.	0.15±0.03[b]	N. D.
C22:2	0.18±0.05[a]	0.36±0.01[a]	1.25±0.93[a]
C20:5	0.68±0.19[c]	1.56±0.34[c]	0.08±0.08[b]
C22:6	24.47±5.92[a]	1.81±0.26[c]	5.29±2.38[b]
**EPA+DHA**	**25.15±6.11**	**3.37±0.6**	**5.37±2.46**
**PUFA**	**29.92±7.21[a]**	**18.57±2.55[b]**	**17.46±9.74[a]**
**TFA**	**76.75±19.55[a]**	**74.42±7.08[a]**	**80.1±37.83[a]**

N. D., not detected.

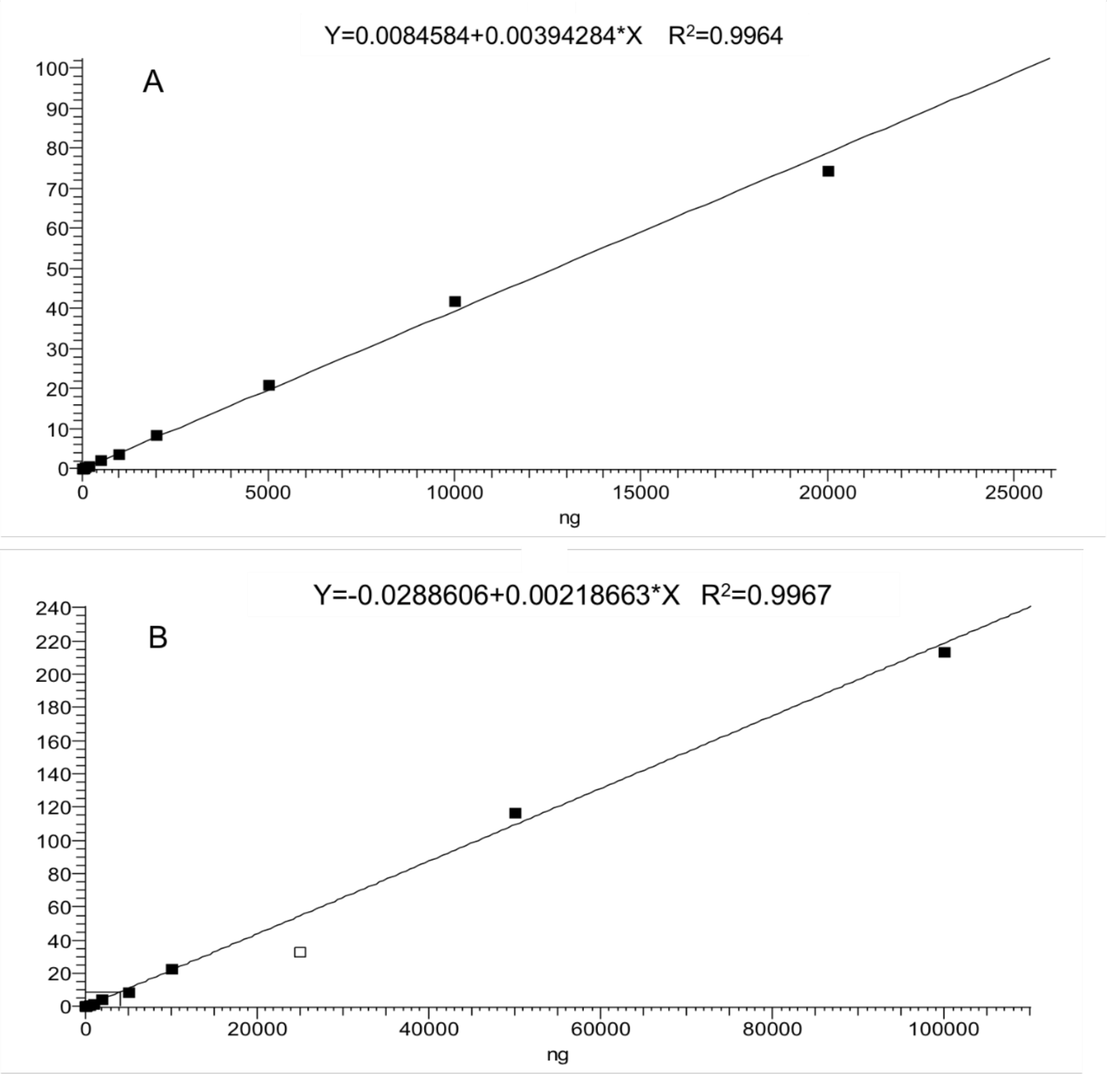

Figure S1. (A) Calibration curve of TG from big eye tuna head using TG (18:/18:1/18:0) as external standard and TG (16:0/18:0/16:0)-d5 as the internal standard, a total of 13 points were prepared to cover the peak area ratio of internal standard and external standard, injection volume is 1μL. (B) Calibration curve of TG from Atlantic salmon and big head carp heads using TG (18:/18:1/18:0) as external standard and TG (16:0/18:0/16:0)-d5 as the internal standard, a total of 13 points were prepared to cover the peak area ratio of internal standard and external standard, injection volume is 1μL.
