# Supplementary figures and images for: Comparison of the Fatty Acid and Triglyceride Profiles of Big Eye Tuna (Thunnus obesus), Atlantic salmon (Salmo salar) and Bighead Carp (Aristichthysnobilis) Heads
Source: Molecules. 2019 Nov 4;24(21):3983. doi: 10.3390/molecules24213983 (PMC6864674; doi:10.3390/molecules24213983)

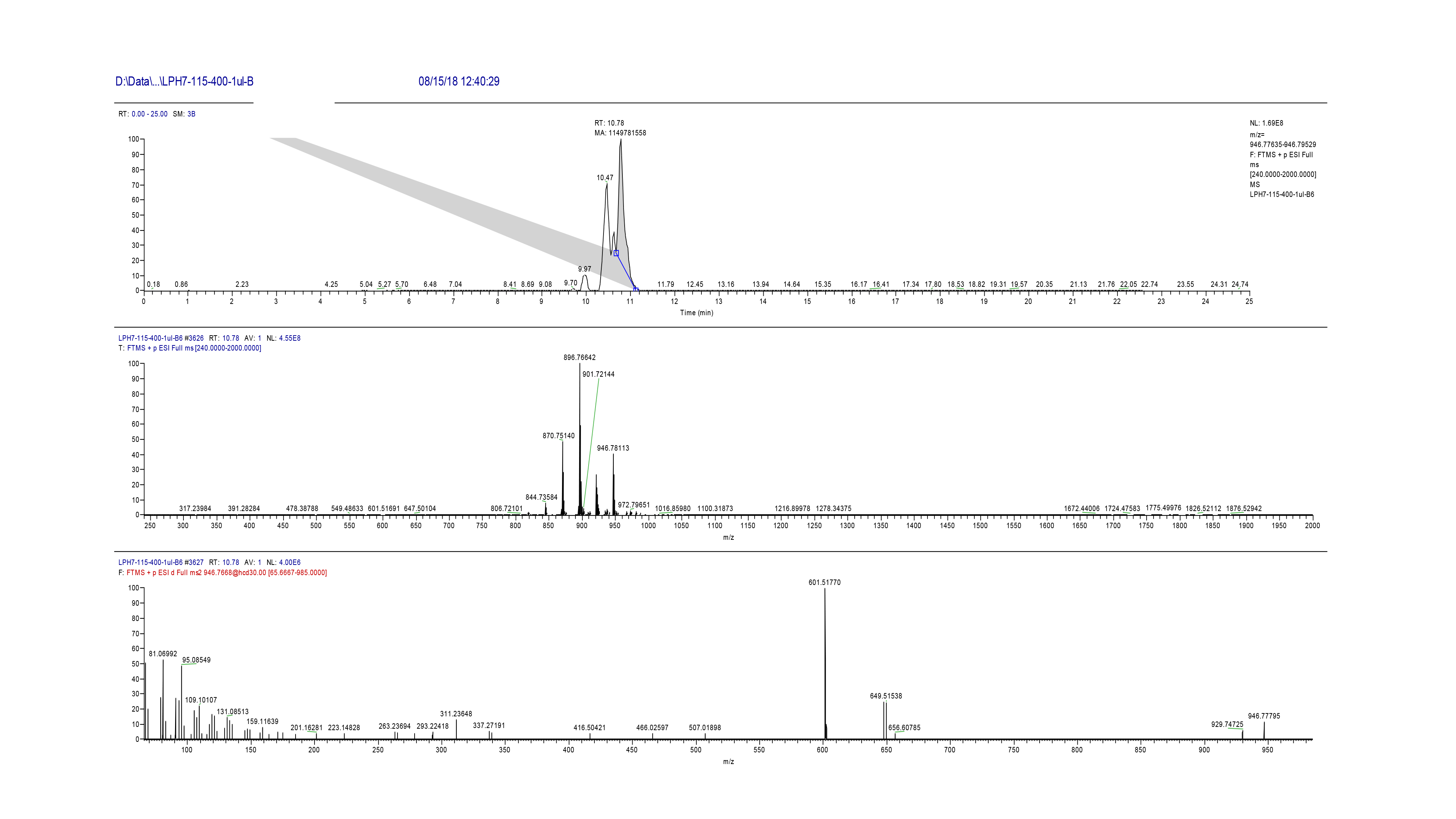

Supplement: Supplementary file 1 [file molecules-24-03983-s001.zip › Supplementary materials/MS and MSMS Spectra of Triglyceride TG 181182226.jpg]

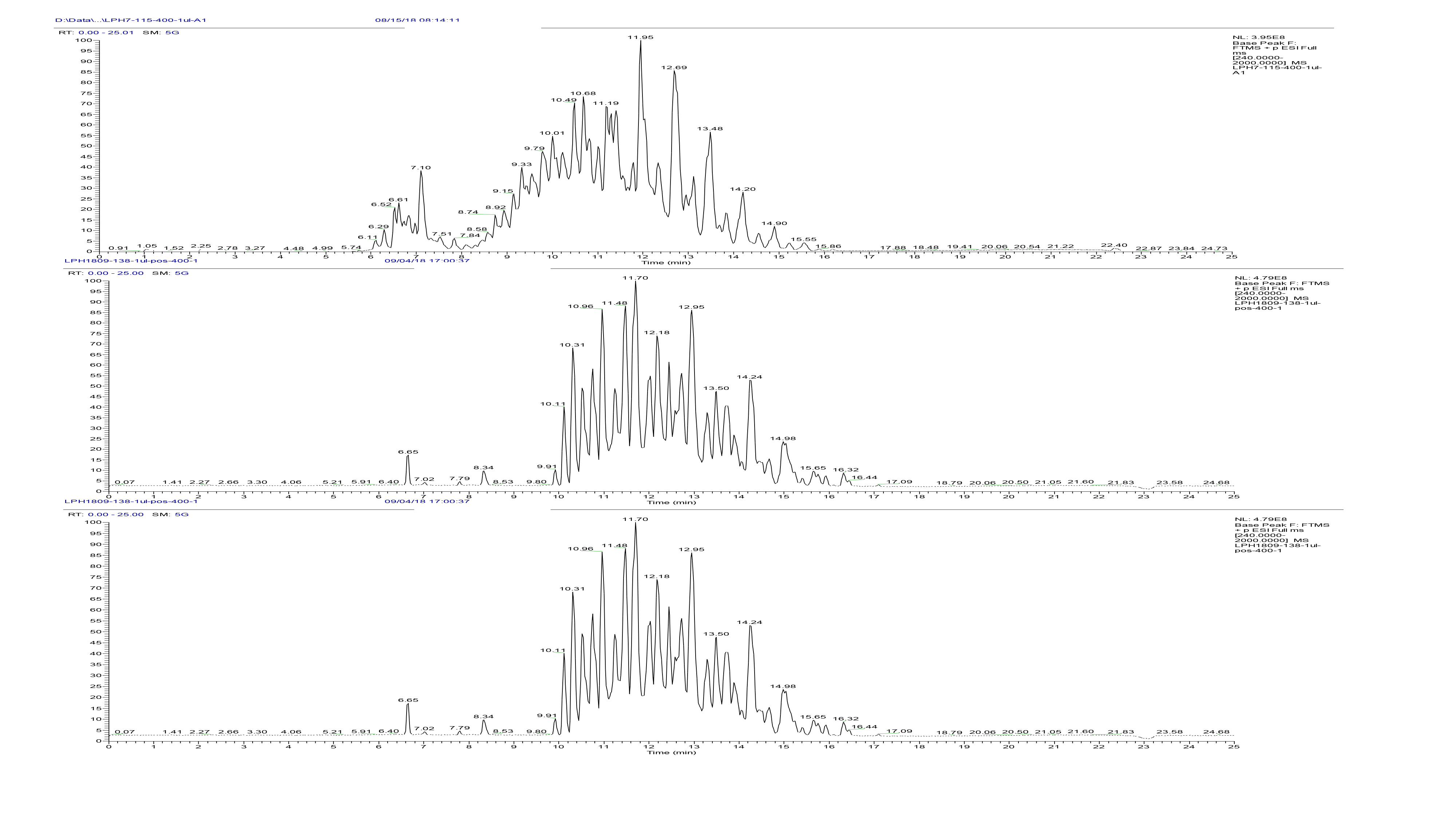

Supplement: Supplementary file 1 [file molecules-24-03983-s001.zip › Supplementary materials/Total ion chromatogram of three kinds of fish heads.jpg]
